# A human factors intervention in a hospital - evaluating the outcome of a TeamSTEPPS program in a surgical ward

**DOI:** 10.1186/s12913-021-06071-6

**Published:** 2021-02-03

**Authors:** Oddveig Reiersdal Aaberg, Marie Louise Hall-Lord, Sissel Iren Eikeland Husebø, Randi Ballangrud

**Affiliations:** 1 Department of Health and Nursing Science, Faculty of Health and Sport Sciences, University of Agder, Universitetsveien 25 A, 4630 Kristiansand, Norge; 2 Department of Health Science, Faculty of Medicine and Health Sciences, Norwegian University of Science and Technology, Teknologivegen 22, 2815 Gjøvik, Norway; 3 Department of Quality and Health Technology, Faculty of Health Sciences, University of Stavanger, Kjell Arholmsgate 41, 4036 Stavanger, Norway; 4 Department of Health Sciences, Faculty of Health, Science and Technology, Karlstad University, Universitetsgatan 2, 651 88 Karlstad, Sweden; 5 Research Group of Nursing and Health Care Sciences, Stavanger University Hospital, Gerd-Ragna Bloch Thorsens gate 8, 4011 Stavanger, Norway

**Keywords:** Human factors, Implementation, Intervention, Interprofessional teamwork, Longitudinal, Patient safety culture, SEIPS 2.0, TeamSTEPPS, Team training

## Abstract

**Background:**

Patient safety in hospitals is being jeopardized, since too many patients experience adverse events. Most of these adverse events arise from human factors, such as inefficient teamwork and communication failures, and the incidence of adverse events is greatest in the surgical area. Previous research has shown the effect of team training on patient safety culture and on different areas of teamwork. Limited research has investigated teamwork in surgical wards. The aim of this study was to evaluate the professional and organizational outcomes of a team training intervention among healthcare professionals in a surgical ward after 6 and 12 months. Systems Engineering Initiative for Patient Safety 2.0 was used as a conceptual framework for the study.

**Methods:**

This study had a pre-post design with measurements at baseline and after 6 and 12 months of intervention. The intervention was conducted in a urology and gastrointestinal surgery ward in Norway, and the study site was selected based on convenience and the leaders’ willingness to participate in the project. Survey data from healthcare professionals were used to evaluate the intervention. The organizational outcomes were measured by the unit-based sections of the Hospital Survey of Patient Safety Culture Questionnaire, and professional outcomes were measured by the TeamSTEPPS Teamwork Perceptions Questionnaire and the Collaboration and Satisfaction about Care Decisions in Teams Questionnaire. A paired t-test, a Wilcoxon signed-rank test, a generalized linear mixed model and linear regression analysis were used to analyze the data.

**Results:**

After 6 months, improvements were found in organizational outcomes in two patient safety dimensions. After 12 months, improvements were found in both organizational and professional outcomes, and these improvements occurred in three patient safety culture dimensions and in three teamwork dimensions. Furthermore, the results showed that one of the significant improved teamwork dimensions “Mutual Support” was associated with the Patient Safety Grade, after 12 months of intervention.

**Conclusion:**

These results demonstrate that the team training program had effect after 12 months of intervention. Future studies with larger sample sizes and stronger study designs are necessary to examine the causal effect of a team training intervention in this context.

**Trial registration number:**

ISRCTN13997367 (retrospectively registered).

## Background

Patient safety in hospitals is being jeopardized, since too many patients experience adverse events [1, 2]. The risk of adverse events in surgical care is higher than in other areas of hospitals [3, 4]. Most adverse events arise not from the solitary actions of individuals but from the systems of which they are a part and with which they interact [5]. Root cause analyses have revealed that human factors, such as poor teamwork and communication failures, are the underlying factors for the majority of adverse events in hospitals [2, 6]. Focusing on patient safety culture is crucial for minimizing adverse events and improving patient safety [7]. An organization’s patient safety culture is the product of individual and group values, beliefs, attitudes, perceptions, competencies, and patterns of behavior that determine the organization’s commitment to quality and patient safety [8]. Patient safety requires that healthcare professionals have the right competencies and tools to perform their tasks. It is therefore crucial to conduct patient safety interventions that focus on healthcare professionals and work system factors that contribute to safe care [9]. In this study, we conducted a team training intervention in a surgical ward.

The surgical ward is a microsystem within a hospital organization and a unit with a high degree of complexity [10]. The interdependency among healthcare professionals contributes to this complexity [1]. Clinical work requires a broad spectrum of competencies, and healthcare professionals are often working under high time pressure [11]. Surgical ward physicians are often called to the operating room for surgical procedures during a work shift [12], and this makes interprofessional teamwork in the wards extra challenging.

Human factors is a multidisciplinary science at the intersection of psychology and engineering [13] and is commonly described as a discipline devoted to studying and improving the interactions among humans and other elements of a system [14]. Human factors interventions aim to improve system performance and prevent accidental harm, which for healthcare means supporting the cognitive and physical work of healthcare professionals and promoting high-quality, safe care for patients [15]. Human factors interventions, such as team training, are regarded as an innovative approach for improving patient safety [16–18]. Team training is described as applying a set of instructional strategies that rely on well-tested tools (e.g., simulation, lectures, and videos) to achieve specific team competencies [19, 20].

Previous research on team training interventions has shown improvements in different areas of teamwork [21, 22] and safety culture [23, 24], reductions in surgical harm [25], and reductions in surgical mortality [26]. However, most of the team training research has been conducted in specialty units, and limited research has investigated teamwork in surgical wards [27] or investigated teamwork over long time frames [28]. Few studies have examined the associations between perceptions of teamwork and patient safety culture after a 12-month team training intervention. Observational studies have found that interprofessional teamwork was associated with organizational culture [29] and that event reporting, communication, and leadership were predictors of patient safety culture [30].

In this study, we implemented Team Strategies and Tools to Enhance Performance and Patient Safety (TeamSTEPPS®) in a surgical ward. TeamSTEPPS is a generic program based on research [31, 32] and is built on five key principles: “Team Structure” and the four team competencies “Leadership”, “Situation monitoring”, “Mutual support” and “Communication” [32]. The four team competencies of TeamSTEPPS have 15 associated tools and strategies that are meant to be implemented in clinical practice to improve performance and patient safety [33]. “Team decision making” is an additional team competency or team process [2, 34, 35] that is not included in the TeamSTEPPS program but was included in this study since it is an important aspect of teamwork and has significance for patient safety and patient care [34, 36]. Research from other areas of hospitals shows that most clinical decisions are still made independently by medical professionals, with only some sharing of information, and that such decisions are rarely made collectively by the interprofessional care team [37].

Since the need to implement team training programs in the surgical ward context is being increasingly recognized, an interprofessional TeamSTEPPS intervention was initiated in a surgical ward. We anticipated that training and implementation of teamwork tools and strategies in daily practice among healthcare professionals would improve professional outcomes in terms of perceptions of teamwork, and organizational outcomes in terms of patient safety culture, since the TeamSTEPPS program focuses on both teamwork and patient safety [32]. It takes time to achieve culture change and to embed and sustain new ways of working. Changes that occur in a short time, due to training experience and excitement, may disappear [23]. Therefore, we measured the effect of the intervention 6 and 12 months after initiation.

The aim of the study was to evaluate the professional and organizational outcomes of a team training intervention among healthcare professionals in a surgical ward after 6 and 12 months. The research questions were as follows:

1. Did professional outcome measured by healthcare professionals’ perceptions of teamwork and organizational outcome measured by patient safety culture improve from baseline to 6 and 12 months of intervention?
2. Did patient safety culture related to the intervention vary by profession group or time, demonstrating an effect of the intervention?
3. Were perceptions of teamwork dimensions associated with patient safety culture in the unit after 12 months?

### Conceptual framework

Teamwork and patient safety may be explained on the basis of an input-process-output (IPO) framework that describes the impact of input on process and output, as in classic system theory [20, 34, 38]. The human factors model “The Systems Engineering Initiative for Patient Safety 2.0” (SEIPS 2.0) is an IPO model developed for innovative patient safety research in healthcare [5, 39]. The model emphasizes structural elements in the work system with a person at the center. The person may be represented by patients, healthcare professionals, or healthcare teams - as in this study. The team members perform a range of tasks using various tools and technologies in an internal and external environment and under specific organizational conditions, which all influence the care processes and which in turn influence the outcomes [5, 39]. Unlike most of the IPO models, the SEIPS model differentiates the outcomes in 1) patient outcomes, 2) professional outcomes and 3) organizational outcomes [39]. The interrelatedness of the elements (person, tasks, tools and technology, organization, internal and external environment) within the work system, and among the work system, process and outcome illustrates the complexity of the system [39].

**Fig. 1:**
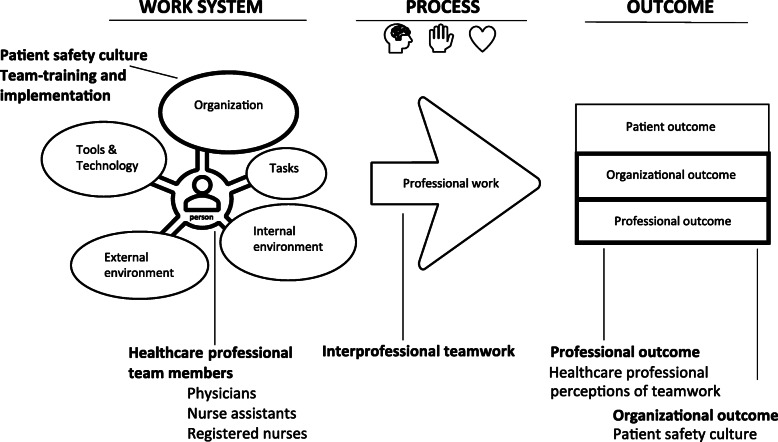
A modified SEIPS 2.0 model adapted from Holden et al. [39]. The components with the bold lines illustrate the input and outcome in this study from a human factors system perspective

In this study, we used the SEIPS 2.0 model to conceptualize the intervention and the outcomes of the study from a system perspective [40]. Implementation of a team training program was regarded as an input in the organization element to strengthen the work system by attempting to improve healthcare professionals’ team competencies and patient safety culture [20, 38]. The SEIPS 2.0 model illustrates how input, in the work system, such as team training, may improve healthcare professionals’ team competencies and influence work processes that in turn influence professional and organizational outcomes. See Fig. 1.

## Methods

### Study design

We conducted a study with a pre-post design with measurements at baseline, after 6 months and after 12 months of intervention.

### Setting and sample

The intervention was conducted in a 20-bed urology and gastrointestinal surgery ward in a 180-bed hospital in Norway. The study site was selected by convenience and based on the leaders’ willingness to participate in the project, motivated by patient safety incidents in the ward. The profile of the surgical ward is displayed in Table 1. No major changes in the unit profile occurred during the study period, except for changes in leadership positions (which is specified in the text in the intervention section). All of the 43 frontline healthcare professionals (12 physicians, 24 registered nurses, and 7 nursing assistants) were invited to participate in the study. A total of 41 participated in the 6-h initial team training. Normal turnover among nurse staff and physicians caused changes in the sample size.

**Table 1:** Unit profile data

	Baseline	6 months	12 months
**Beds and** ***nurse/bed*** **ratio**			
Number of patient beds	20	20	20
*Nurse/bed* ratio	1.16	1.16	1.16
**Full-time equivalent positions**			
Physicians	13	12	12
Registered nurses	17.25	19.25	20.25
Nursing assistants	4.95	3.1	2.1
Unit nurse director	1.0	1.0	1.0
Clinical nurse specialist	1.0	1.0	1.0
**Change in positions**			
Clinical nurse specialist	–	No	No
Unit nurse manager	–	No	Yes
Physician leader gastrointestinal surgery	–	No	No
Physician leader urology	–	No	Yes
Chair of the surgical department	–	No	Yes
**Patient data and sick leave** (previous 6 months)			
Number of patient admissions per month	192	174	173
Length of stay (mean days)	3.46	3.63	3.62
Occupied beds	87%	96%	89%
Emergency admissions	64%	65%	66%
Sick leave nursing staff	13.22%	5.05%	7.58%
Sick leave physicians	3.55%	1.47%	2.58%
**Registered adverse events by year**	**2015**	**2016**	**2017**
Numbers of reported adverse events	38	42	52

### The intervention

The intervention was conducted according to the TeamSTEPPS implementation plan, which comprises three phases, that are based on Kotters change model [32] and aligns with the Clinical Human Factors Group recommendation for team training interventions [41].

#### Phase 1. Set the stage and decide what to do - assessment and planning

A site assessment was conducted and an overview of TeamSTEPPS was provided to the leadership of the surgical department and the leaders of the selected ward. After the leaders had decided that their unit was ready for the TeamSTEPPS program, an intervention plan was developed jointly by a project group consisting of the researchers and the leaders of the ward. The leaders consisted of the chair of the surgical department, the unit nurse manager, and the two head surgeons (urology and gastrointestinal surgery). In advance of the intervention start, the physicians and nursing staff attended information meetings conducted by the researchers.

#### Phase 2. Make it happen - training, planning and implementation

The onset of the intervention was a mandatory 6-hour interprofessional TeamSTEPPS training distributed over 3 days in a period of 3 weeks. In advance of the training, TeamSTEPPS leaflets and pocket-guides were distributed to all healthcare personnel, which they were asked to read in preparation for the training. The training was conducted in a simulation center at a university and delivered by the master trained nurses and physician leaders in the surgical ward. The team training was a combination of didactics, videos, role play and high-fidelity simulation training. The simulation training included debriefing sessions with a focus on interprofessional teamwork. The first lecture, held by the chair of the surgical department, aimed to create a sense of urgency by presenting the hospital’s reports of adverse events. At the end of the training, the healthcare professionals were asked to identify patient safety issues in the ward and to suggest TeamSTEPPS tools to solve the problems. Immediately after the training, the participants responded to the “The TeamSTEPPS Course Evaluation Survey”. The evaluation results were very good, both regarding training satisfaction and learning outcomes [42].

After the training, an interprofessional change team was established. The change team consisted of 12 members representing all levels in the organization, including a former patient and one of the researchers (ORA), and it was led by the unit nurse manager. The researcher coached the change team. Based on the identified safety issues, the change team developed an action plan, according to which they implemented tools and strategies into daily practice. The vision of the action plan was “Zero errors”, and the specific goals were aligned with the organizational goals of the surgical department. The unit nurse manager, the clinical nurse specialist, and the two head surgeons, led the implementation in collaboration with the other members of the change team.

Five tools were implemented in the ward during the first 6 months of the study period, at a rate of approximately one tool per month (Table 3). The tool of the month was communicated through weekly newsletters and staff meetings and implemented in daily practice. A description of the selected tools and strategies implemented in the ward is displayed in Table 2, and an overview of the start times of a new tool to be implemented is displayed in Table 3. Refresher training for the nursing staff (75 min), and for physicians (20 min) were conducted 5 months after the initial team training.

**Table 2:** Explanation of the selected tools and strategies implemented in study period [32]

TeamSTEPPS tools and strategies	Explanation
**Closed-loop**	Using closed-loop communication to ensure that information conveyed by the sender is understood by the receiver as intended
**ISBAR**	A technique for communicating critical information that requires immediate attention and action concerning a patient’s condition
**I-PASS**	Strategy designed to enhance information exchange during transitions in care
**Brief**	Short session prior to start to share the plan, discuss team formation, assign roles and responsibilities, establish expectations and climate, anticipate outcomes and likely contingencies
**Huddle**	Ad hoc meeting to re-establish situational awareness, reinforce plans already in place, and assess the need to adjust the plan
**Debrief**	Informal information exchange session designed to improve team performance and effectiveness through lessons learned and reinforcement of positive behaviors
**Task assistance**	Helping others with tasks builds a strong team. Key strategies include: Team members protect each other from work overload situations, Effective teams place all offers and requests for assistance in the context of patient safety, Team members foster a climate where it is expected that assistance will be actively sought and offered
**The two- challenge rule**	Empowers all team members to “stop the line” if they sense or discover an essential safety breach. When an initial assertive statement is ignored: It is your responsibility to assertively voice concern at least two times to ensure that it has been heard, The team member being challenged must acknowledge that concern has been heard, If the safety issue still hasn’t been addressed: Take a stronger course of action; Utilize supervisor or chain of command
**Cross monitoring**	A harm error reduction strategy that involves: Monitoring actions of other team members, Providing a safety net within the team, Ensuring that mistakes or oversights are caught quickly and easily, “Watching each other’s back”
**STEP**	Tool to help assess health care delivery situations

**Table 3:** Time of implementation of the selected TeamSTEPPS tools and strategies

The teamwork competencies	May 2016	June 2016	August 2016	September 2016	October 2016	January 2017	February 2017	March 2017	May 2017
**Communication**	Closed-loop	ISBAR^1^							I-PASS^3^
**Leadership**			Briefs	Huddles		Debriefs			
**Situation Monitoring**					Cross monitoring		STEP^2^		
**Mutual Support**						Task assistance		Two Challenge rule	

^1^ISBAR = Identification, Situation, Background, Assessment, Request/Recommendation – Use by exchange of critical information

^2^STEP=Status of the patient, Team members, Environment, Progress toward the goal – Used by focusing on updated electronic care plans

^3^I-PASS=Illness severity, Patient summary, Action list, Situation awareness and contingency planning, Synthesis by receiver – Systematic handoffs with focus on patient safety risks

After 8 months of intervention, some changes in the wards’ leadership occurred. The master trained head surgeon of urology left employment at the hospital. The chair of the department moved to a higher position in the hospital organization, and the head surgeon of the gastrointestinal surgery section assumed the position of chair. The unit nurse manager was allocated to a position as assistant chair of the surgical department, and the clinical nurse specialist assumed the role of the leader of the change team (Table 1).

#### Phase 3. Make it stick – sustainment

Rather than reducing the intervention pressure, it was maintained, and the implementation of tools and strategies continued. Five more tools were implemented during the last 6 months of the 12-month study period (Table 3). Achievements were celebrated along the way. When conducting whiteboard patient safety huddles after rounding every day, 30 days in a row, they celebrated with a whiteboard-themed cake.

After 11 months, another refresher training session was held for the nursing staff (75 min), but not for the physicians (due to busy work schedules). Other than the missed refresher training, the intervention was conducted as intended, with the interprofessional change team and leadership leading the change, and with a project group that had meetings every second month throughout the project period [43].

### Measurements

Three questionnaires were used to evaluate the intervention. For measuring the professional outcomes (teamwork), the TeamSTEPPS Teamwork Perceptions Questionnaire (T-TPQ) and the Collaboration and Satisfaction about Care Decisions in Teams (CSACD-T) were used, and for measuring organizational outcomes (patient safety culture), the Hospital Survey of Patient Safety Culture Questionnaire (HSOPS) was used.

The T-TPQ is a 35-item questionnaire [44, 45] that measures individuals’ perception of the level of teamwork that exists in their work unit. Participants responded using a 5-point Likert scale of agreement (5 = strongly agree to 3 = neutral to 1 = strongly disagree). The T-TPQ measures five teamwork dimensions addressed in the TeamSTEPPS program; there are seven items for each of the following five dimensions: “Team structure”, “Leadership”, “Mutual Support”, “Situational Monitoring” and “Communication”.

The CSACD-T is a questionnaire measuring clinical decision making in teams. It is composed of seven items with statements regarding collaboration in team decision making about patient care and two items about satisfaction with decision making. The participants responded by using a 7-point Likert scale of agreement (from 1 = strongly disagree to 7 = strongly agree), global collaboration (from 1 = no collaboration to 7 = complete collaboration), and satisfaction about care decisions (from 1 = not satisfied to 7 = very satisfied). The questionnaire was developed from the original nurse-physician “Collaboration and Satisfaction about Care Decisions” questionnaire [46].

The HSOPS [47] is a questionnaire that assesses the extent to which healthcare professionals’ organizational culture supports patient safety. It is recommended for evaluating the cultural impact of team training and patient safety interventions [47]. The full HSOPS comprises 2 single items and 12 patient safety culture dimensions. Each dimension is composed of three or four items [47]. The two single items (“Number of Events Reported” and “Patient Safety Grade”) and two of the dimensions (“Overall Perceptions of Patient Safety” and “Frequency of Events Reported”) are regarded as outcome measures. Three dimensions are regarded as hospital-level measures [48]. Because we only studied one unit, we excluded the hospital-level section of the questionnaire (11 items – 3 dimensions) and used the 2 single items and the remaining 33 items of the nine unit-level dimensions: “Teamwork Within Unit”, “Manager’s Expectations & Actions Promoting Patient Safety”, “Organizational Learning - Continuous Improvement”, “Feedback and Communication About Error”, “Communication Openness”, “Staffing”, “Nonpunitive Response to Errors”, “Overall Perceptions of Patient Safety”, and “Frequency of Events Reported” [48]. The participants responded by using a 5-point Likert scale of agreement (from 1 = strongly disagree to 5 = strongly agree, with “neither” in the middle) or frequency (from 1 = very seldom to 5 = very often). The single item “Patient Safety Grade”, which asks participants to provide an overall grade on patient safety for their unit, has the following five response options: A = Excellent, B = Very Good, C = Acceptable, D = Poor, E = Failing. The single item “Number of Events Reported”, which indicates the number of adverse events the participants have reported over the past 12 months, has six response options: 1 = No events, 2 = 1 to 2 events, 3 = 3 to 5 events, 4 = 6 to 10 events, 5 = 11 to 20 events, 6 = 21 events or more [47].

All three questionnaires were translated into Norwegian and psychometrically tested [49–51]. In addition to the questionnaires, participants’ background information was solicited (sex, age group, profession group, and employee time in the unit).

### Data collection

An electronic survey (SurveyXact) was distributed by email to the healthcare professionals to evaluate the effect of the TeamSTEPPS program. Data collection was conducted at baseline (February–March 2016) and after 6 months (November–December 2016) and 12 months of intervention (June 2017). Unit profile data were collected from the unit nurse manager.

### Statistical analyses

To test for statistically significant changes between baseline and 6 months and between baseline and 12 months, a paired t-test was applied on the healthcare professional’s mean scores of the T-TPQ and HSOPS dimensions and the total score of the CSACD-T, and a Wilcoxon signed-rank test was applied on the two single items of the HSOPS [52]. A generalized linear mixed model (GLMM) [53] was used to investigate the outcome of TeamSTEPPS by estimating the associations among the nine HSOPS dimensions used as dependent variables and “Profession group” (nursing staff and physicians) and “Time” (baseline, after 6 and 12 months of intervention) as the two independent variables. A GLMM is a generalization of traditional linear regression that adjusts for the correlation between repeated measurements within each subject and finds the best linear fit to the data across all individuals. The model maximizes power by utilizing all data despite missing observations in some subjects [54, 55]. The GLMM was applied to the total sample (*n* = 98), and the results are reported as estimates with 95% confidence intervals. To test whether any of the three significant improved teamwork dimensions of the T-TPQ were associated with two of the patient safety culture outcomes (“Overall patient safety” and “Patient Safety Grade”) after 12 months of intervention, multiple linear regression analysis was performed on all healthcare professionals (*n* = 31) who responded after 12 months of intervention [56]. A *p*-value < .05 was considered to be statistically significant for all analyses. Statistical Package for Social Sciences (SPSS) version 24 (Armonk, New York) and R 3.1.1 were used to analyze the data. The study adheres to the Transparent Reporting of Evaluations with Nonrandomized Designs (TREND) guidelines [57].

## Results

Of the 43 invited healthcare professionals in the ward, 35 of them responded to the survey at baseline. After 6 months of the intervention, 32 healthcare professionals responded, of which 28 had also responded at baseline. After 12 months of the intervention, 31 healthcare professionals responded, of which 25 had responded at baseline. A total of 98 responses from all respondents were collected at the three time points. See Table 4 for an overview. The characteristics of the respondents are displayed in Table 5.

**Table 4:** Samples and respondents

	Sample	n	Response rate
Baseline	43	35	81%
After 6 months of intervention	42	32	76%
After 12 months of intervention	40	31	78%
In total		98	
Both baseline and after 6 months		28	
Both baseline and after 12 months		25

**Table 5:** Characteristics of the respondents

	***n*** = 28 6 months	***n*** = 25 12 months
	n (%)	n (%)
**Gender**		
Female	23 (82)	22 (88)
Male	5 (18)	3 (12)
**Profession**		
Physicians	6 (21)	4 (16)
Assistant nurses	4 (14)	3 (12)
Registered nurses	18 (64)	18 (72)
**Age**		
≤ 30 years	6 (22)	4 (16)
31–50 years	12 (44)	12 (48)
≥ 51 years	9 (33)	9 (36)
Missing	1	
**Time employed in the unit**		
0–5 years	6 (25)	2 (8)
6–15 years	11 (46)	12 (50)
≥ 16 years	7 (29)	10 (42)
Missing	4	1

The mean scores on the T-TPQ, CSACD-T and HSOPS for those answered two times (baseline and after 6 months or baseline and after 12 months) are displayed in Table 6. None of the teamwork dimensions of the T-TPQ showed significant changes after 6 months. After 12 months of intervention, significant improvements were found in three teamwork dimensions, regarded as professional outcomes: “Situation Monitoring”, “Mutual Support”, and “Communication”. No significant changes were found in the professional outcome “Team decision making” (CSACD-T) during the study period.

**Table 6:** Healthcare professional perceptions of teamwork and patient safety culture from baseline to 6 and 12 months of intervention

	*n* = 28				n = 25			
	baseline mean	6 months mean	change from baseline to 6 months		baseline mean	12 months mean	change from baseline to 12 months	
			t^**1**^	p^**1**^			t^**1**^	p^**1**^
**T-TPQ**^**2**^ **dimensions**								
Team Function	3.93 (.40)	3.96 (.44)	.48	.638	3.95 (.43)	4.08 (.44)	1.71	.100
Leadership	4.24 (.40)	4.21 (.49)	−.39	.700	4.16 (.39)	4.15 (.63)	−.09	.926
Situation Monitoring	3.79 (.47)	3.98 (.56)	1.74	.094	3.70 (.43)	4.06 (.54)	4.70	**.001**
Mutual Support	3.85 (.44)	3.93 (.51)	.89	.382	3.83 (.44)	4.03 (.50)	1.04	**.027**
Communication	3.84 (.40)	3.94 (.50)	3.34	.345	3.81 (.39)	4.02 (.53)	2.66	**.015**
**CSACD-T**^**3**^								
Team Decision Making	4.73 (.89)	5.02 (1.09)	1.29	.207	4.69 (.92)	4.95(1.03)	1.32	.200
**HSOPS**^**4**^ **dimensions**								
Teamwork Within Unit	3.87 (.54)	4.08 (.52)	1.80	.084	3.78 (.52)	4.05 (.51)	2.39	**.025**
Manager Expect. & Actions Promoting Pat. Safety	4.18 (.60)	4.29 (.50)	.91	.370	4.11 (.56)	4.39 (.52)	2.72	**.012**
Organizational Learning – Cont. Improvement	3.82 (.51)	4.05 (.61)	1.8	**.001**	3.76 (.51)	3.97 (.65)	1.78	.087
Feedback & Communication About Error	3.71 (.62)	3.85 (.70)	.04	.965	3.65 (.58)	3.90 (.60)	1.84	.078
Communication Openness	3.83 (.49)	4.07 (.60)	2.37	**.025**	3.77 (.59)	3.97 (.49)	2.58	**.017**
Staffing	3.52 (.46)	3.39 (.52)	−1.08	.292	3.81 (.49)	4.07 (.53)	.06	.955
Nonpunitive Response to Errors	2.90 (.69)	3.14 (.83)	1.38	.178	2.86 (.66)	3.01 (.84)	.97	.342
Frequency of Events Reported^5^	2.88 (.70)	3.13 (.84)	1.98	.059	3.49 (.45)	3.50 (.66)	1.09	.287
Overall Perceptions of Patient Safety^5^	4.12 (.51)	4.28 (.50)	.90	.375	4.13 (.49)	4.27 (.62)	1.94	.065
**HSOPS**^**4**^ **single items**			**z-score**^**6**^	**p**^**6**^			**z-score**^**6**^	**p**^**6**^
Number of Events Reported^5^	2.11 (.83)	2.00 (.80)	−.63	.527	2.24 (.78)	2.15 (.72)	−.78	.439
Patient Safety Grade^5^	3.67 (.56)	3.79 (.59)	−.82	.414	3.67 (.57)	3.92 (.56)	−.1.9	.059

^1^Paired t-test

^2^T-TPQ = TeamSTEPPS Teamwork Perceptions Questionnaire (scale 1–5)

^3^CSACD-T = Collaboration and Satisfaction About Care Decisions in Teams Questionnaire (scale 1–7)

^4^HSOPS = Hospital Survey of Patient Safety Culture Questionnaire (scale 1–5)

^5^Patient Safety outcome measures

^6^Wilcoxon Signed Ranks Test

The patient safety culture results (HSOPS), regarded as organizational outcomes, showed significantly improved scores in two dimensions after 6 months of intervention: “Organizational Learning & Continuous Improvement” and “Communication Openness”. The three dimensions “Communication Openness”, “Teamwork Within Unit” and “Manager’s Expectations & Actions Promoting Patient Safety” were significantly improved after 12 months.

The results of the GLMM estimates of organizational outcome (patient safety culture outcome) showed that both ‘Organizational Learning and Continuous Improvement’ and ‘Communication Openness’ had a significant effect after 6 months. Overall, physicians had a significant positive, as effect compared to nursing staff, on both ‘Frequency of Events Reported’ and ‘Patient Safety Grade” (

**Table 7:** Estimated Patient Safety Culture by “Time” and “Profession group” (*n* = 98)

Parameter	Estimate	95% Confidence Interval	***p***^**1**^
**Organizational Learning and Continuous Improvement**			
Intercept	3.80	3.60, 4.00	.000
Baseline^2^	0^b^		
6 months of intervention	.33	.05, .60	**.020**
12 months of intervention	.18	-.09, .46	.193
Nursing staff^2^	0^b^		
Physicians	-.27	-.54, .00	.051
**Communication Openness**			
Intercept	3.80	3.63, 4,02	.000
Baseline^2^	0^b^		
6 months of intervention	.29	.02, .55	**.035**
12 months of intervention	.21	-.05, .48	.116
Nursing staff^2^	0^b^		
Physicians	-.12	-.38, .14	.366
**Frequency of Events Reported**			
Intercept	2.73	2.46, 3.00	.000
Baseline^2^	0^b^		
6 months of intervention	.26	-.11, .63	.164
12 months of intervention	.13	-.25, .51	.500
Nursing staff^2^	0^b^		
Physicians	.56	.19, .93	**.003**
**Patient Safety Grade**			
Intercept	3.60	3.41, 3.79	.000
Baseline^2^	0^b^		
6 months of intervention	.11	-.16, .38	.410
12 months of intervention	.25	-.02, .52	.074
Nursing staff^2^	0^b^		
Physicians	.40	.14, .66	**.003**

Table 7).

The multiple linear regression analysis of all respondents after 12 months (*n* = 31) found that the three improved teamwork dimensions “Situational Monitoring”, “Mutual Support” and “Communication” (independent variables) explained 31.6% of the variance in the “Patient Safety Grade” after 12 months of intervention. The model reached statistical significance (*p* = .012). When analyzing which of the three independent variables contributed to the prediction of “Patient Safety Grade”, the model showed that “Mutual Support” had the largest ß coefficient (ß = .76) and that the effect was significant (*p* = .036). When testing with the “Overall Perceptions of Patient Safety” as the dependent variable, the model reached statistical significance (*p* = .021). The three teamwork dimensions explained 24.3% of the variance in the “Overall Perceptions of Patient Safety” after 12 months of intervention but with a low ß-coefficient and without statistical significance.

## Discussion

Regarding organizational outcomes as related to the SEIPS 2.0 model, improvements were found in two patient safety culture dimensions after the first 6 months of this comprehensive intervention. No improvement was found in professional outcome after the first 6 months, as measured by perceptions of teamwork. After the full 12 months, however, improvements were found in both professional and organizational outcomes. Improvement in professional outcomes were shown in three out of four perceptions of teamwork dimensions. Regarding organizational outcomes, improvements were found in three patient safety culture dimensions. These results indicate that the team training program had an effect after 12 months of implementation. The GLMM estimates demonstrated an effect of time on the patient safety culture dimensions (organizational outcome) “Organizational Learning and Continuous Improvement” and “Communication Openness” after 6 months, and the estimates also demonstrated that physicians had an overall positive significant effect compared to nursing staff on the patient safety culture dimensions “Frequency of Events Reported” and “Patient Safety Grade”. Furthermore, the teamwork dimension “Mutual Support” was associated with “Patient Safety Grade” after 12 months of intervention.

No significant improvement after 6 months in T-TPQ measures may be explained by the fact that few of the TeamSTEPPS tools had been implemented by that point. However, we expected to find improvement in “Communication” after 6 months since the tools Closed-loop and ISBAR (Identification, Situation, Background, Assessment, Request/Recommendation) were implemented in the work system in an early phase of the intervention. After 12 months of intervention, however, the results showed improvement in three teamwork dimensions (“Situation Monitoring”, “Mutual Support”, and “Communication”). The cross-monitoring strategy was implemented after 5 months, and the STEP (Status of the patient, Team members, Environment, Progress toward the goal) tool was implemented after 9 months [58], so the improvement in “Situation Monitoring” may be due to the implementation of these tools. “Situation Monitoring” involves continuously scanning the environment for important information, watching out for other team members, exchanging relevant information, and jointly reevaluating patient goals [44]. The improved scores in “Mutual Support” may be a result of the “Task Assistance” and “Two Challenge Rule” strategies that were implemented in the work system during the study period [58]. “Mutual Support” is about cautioning each other about potentially risky patient safety situations and about assisting one another during high workloads [44]. When observing these improvements in teamwork dimensions from a system perspective, they are seen as improved professional outcomes (see Fig. 1). Previous studies from the context of surgical wards that have measured self-reported teamwork have produced ambiguous results [59–61]. Paull, DeLeeuw [61] found improvement in all scores in their multicenter study when the scores were measured immediately after the training. Study results collected a short time after a team training may benefit from the positive experience the participants have just had and can be seen to reflect a strong Hawthorne effect [62]. The reason why we did not see improvements in team decision making in our study may be due to the time points selected for measurement. Previous studies that showed enhanced scores in decision making measured 2 weeks and 2 months after simulation training [63, 64]. Our results for team decision making may also be explained by the fact that the TeamSTEPPS program does not emphasize decision making, and therefore, there was not a focus on this important aspect of teamwork in the intervention. In the teamwork literature from Europe, where team competencies are referred to as team skills, decision making is one of the six skills in the definition of non-technical skills (NTS) [65]. Furthermore, decision making has also recently been emphasized in the teamwork literature, indicating significance for patient safety and patient outcomes [2, 34, 35].

The organizational outcome measured by patient safety culture showed improvement in “Organizational Learning & Continuous Improvement” and “Communication Openness” after 6 months of intervention, and improvement in the latter was sustained after 12 months, both of which are interesting results. “Communication Openness” is a measure of whether staff freely speak up if they see something that may negatively affect a patient and if they feel free to question those with more authority than themselves [66]. This result is therefore of importance regarding the patient safety culture in the ward, as it may contribute to catching adverse events before it reaches a patient. Regarding whether the healthcare professionals reported diverse types of adverse events in our study, the average answer was “sometimes” at all data collection times, while the registered adverse events increased during the study period. An increase in adverse events is not desirable, but may be seen as an improvement in the reporting culture. The main purpose of reporting is to learn from adverse events [67], and learning is an important part of the human factors approach to patient safety. After 6 months, improvements were found in organizational outcomes (in two patient safety dimensions). After the full 12 months, improvements were found in both organizational outcomes (three patient safety culture dimensions) and professional outcomes (three teamwork dimensions). The mixed model estimates demonstrated that physicians had effects on two patient safety culture measures. Furthermore, the results showed that teamwork was associated with Patient Safety Grade [68]. The improvement in the HSOPS dimension “Organizational Learning – Continuous Improvement” (organizational outcome) may indicate that the healthcare professionals perceived their ward as a learning unit. This result also supports the mixed model estimate, which demonstrated that the time had an effect on “Organizational Learning & Continuous Improvement” after 6 months. The estimates also demonstrated that the healthcare professionals` perceptions of “Communication Openness” were affected by time (6 months), which corresponds with the results from the t-test analyses, where “Communication Openness” showed significant improvements after both 6 and 12 months. The estimates from the mixed models that suggested that physicians had a positive effect on the intervention compared to nursing staff on two patient safety culture dimensions is an interesting finding since it is often challenging to involve physicians in interprofessional interventions in wards [69]. Although we cannot say for sure what caused what, we consider the interprofessional approach to training and implementation as crucial to success in the quality improvement of teamwork and patient safety work in hospital wards. The interprofessional approach may have influenced the professional and organizational outcomes in a positive way. In addition to the sustained improvement in “Communication Openness”, two more dimensions of HSOPS were improved after 12 months: “Teamwork Within Unit” and “Manager’s Expectations & Actions Promoting Patient Safety”. As a part of an enabling work environment, management and leadership are important enablers in achieving effective teamwork and patient safety in complex organizations [70]. This teamwork and patient safety intervention, led by the leaders and the other members of the change team, may have contributed to improvements in these dimensions. The changes in leadership positions may also have accounted for the improvement, but this is uncertain. However, although the master trained nurse unit manager resigned from the unit, she continued to work in the administration of the department and continued to give support and guidance for the intervention from her new position.

Our improved patient safety culture results in three dimensions of the HSOPS (organizational outcome) are in line with those from previous research in diverse hospital contexts. Two multicenter studies found improvement in three HSOPS dimensions when measured after 12 months [71, 72], and Thomas and Galla [69] found improvements in three HSOPS dimensions after 2 years. Schwartz, Welsh [72] found a decrease from 6 to 12 months in their multicenter study, a decrease they explained with a need for early refresher training.

The improved professional outcome “Mutual Support” was associated with “Patient Safety Grade” at the end of the study period, which is interesting from a human factors perspective since this T-TPQ dimension Mutual Support encompasses items focus on patient safety and emphasizes the strong patient safety aspect of the TeamSTEPPS program.

The use of the conceptual framework contributed to an enhanced understanding of the system approach in our study, which is important to implement and sustain innovations [73]. When implementing teamwork tools, such as ISBAR, Closed-loop, and Cross-monitoring [58] in the work system, the use of the tools and strategies in the clinical work processes have influenced professional outcomes indicating that the teamwork competencies of the healthcare professionals improved during the study period. Transfer of the learning from team training is crucial to patient safety and interesting from a human factors perspective, as outcomes are influenced by the learning-to-transfer pathway [74]. The improvement in organizational outcomes (patient safety culture) may be due to the TeamSTEPPS intervention in the work system (see Fig. 1).

The implementation of teamwork tools that initiated new ways of working may in time lead to system changes, but that was beyond the scope of this study. The healthcare professionals in hospital wards are organized in silos and system changes and structural changes that promote teamwork and patient safety are warranted in the future [1].

### Study limitations

The study has some limitations. The lack of randomization and controls may have threatened the internal validity, although a pre-post design is useful where there are practical barriers to a randomized design [75]. The study samples were small, but the response rates were satisfying, without risk of response bias. Because of the uncontrolled design, we cannot conclude that the improvements were due to the intervention. There are always secular trends that might be occurring at the same time in a surgical ward, and which may have influenced our results [76]. However, because of these study limitations, caution must be taken in generalizing the results.

## Conclusions

This study showed the effect of a human factors team training intervention after 12 months of implementation in a surgical ward, an effect that was demonstrated by both professional and organizational outcomes in the SEIPS 2.0 model. More work needs to be done to investigate the effect of TeamSTEPPS interventions in surgical wards, and studies with larger sample sizes and stronger designs are preferred. Future studies testing the causal pathways identified by SEIPS 2.0 will be of special interest.

## Data Availability

The datasets used during the current study are available from the corresponding author on reasonable request.

## Abbreviations

CSACD-T: Collaboration and Satisfaction with Care Decisions in Teams
GLMM: Generalized Linear Mixed Model
HSOPS: The Hospital Survey of Patient Safety Culture
ISBAR: Identification, Situation, Background, Assessment, Request/Recommendation
SEIPS: Systems Engineering Initiative for Patient Safety
STEP: Status of the patient, Team members, Environment, Progress toward the goal
I-PASS: Illness severity, Patient summary, Action list, Situation awareness and contingency planning, Synthesis by receiver
TeamSTEPPS: Team Strategies and Tools to Enhance Performance and Patient Safety
T-TPQ: TeamSTEPPS Teamwork Perceptions Questionnaire
